# A Novel Ultrasonic Method for Liquid Level Measurement Based on the Balance of Echo Energy

**DOI:** 10.3390/s17040706

**Published:** 2017-03-28

**Authors:** Bin Zhang, Yue-Juan Wei, Wen-Yi Liu, Yan-Jun Zhang, Zong Yao, Liang Zhang, Ji-Jun Xiong

**Affiliations:** 1 Key Laboratory of Instrumentation Science & Dynamic Measurement, Ministry of Education, North University of China, Taiyuan 030051, China; zb0003@126.com (B.Z.); yaozong126@sina.com (Z.Y.); zhangliang_ty@163.com (L.Z.); xiongjijunnuc@126.com (J.-J.X.); 2 Science and Technology on Electronic Test & Measurement Laboratory, North University of China, Taiyuan 030051, China; 3 Software School of North University of China, Taiyuan 030051, China; weiyuejuan@nuc.edu.cn

**Keywords:** ultrasonic impedance, balance, echo energy, coupling, matching layer

## Abstract

This study presents a novel method for determining the liquid level from the outside of a sealed container, which is based on the balance of echo energy received by two receiving sensors. The proposed method uses one transmitting transducer and two receiving sensors that are encapsulated in a coupling plane and arranged by certain rules. The calculation and comparison of echo energy are grounded on the difference ultrasonic impedance between gas and liquid media. First, by analyzing the propagation and attenuation characteristics of ultrasonic waves in a solid, an acoustic model for calculating the echo energy is established and simulated in MATLAB. Second, the proposed method is evaluated through a series of experiments. The difference and ratio of echo energy received by two receiving sensors are calculated and compared under two different coupling conditions. Two kinds of the sensors that are arranged by different rules are selected for measuring the liquid level, and the measurement are analyzed and discussed in detail. Finally, the experimental results indicate that the proposed method can meet the proposed accuracy requirements and can effectively solve the problems caused by some poor coupling conditions.

## 1. Introduction

In fields such as petroleum, chemical, and aerospace, the real-time monitoring and accurate measurement of the liquid level in a sealed container are important for the industrial automation and safety production [1,2]. In actual industrial production, an appropriate measurement method should be selected to suit the specific environment and safety requirements [3,4]. There are several traditional liquid level measurement methods [1,2,3,4,5,6], of which the technologies are stable and reliable and the measurement results are accurate. However, these methods usually require some sensors or all of detection equipment to be installed inside a container in advance. Some special industries require a container to be stored with high temperature, high pressure, inflammable, explosive, highly corrosive, or strong, volatile liquid inside. It is easy for a leakage accident to occur, and after a containment failure, maintenance is difficult and costly.

For these reasons, some scholars and institutions in the United States began in the 1980s to study and use new technologies based on fiber optics, ultrasound, lasers, and gamma rays for liquid level measurement. Among these, ultrasonic detection technology can achieve a true sense of non-contact and non-immersion measurement without damaging the physical structure and integrity of a container. Therefore, ultrasonic technology for liquid level detection has been developed rapidly in recent years.

Generally, the liquid level measurement methods based on ultrasonic technology can be divided into four types according to the realization principle, as described in literature [1]. All of them have strengths of ultrasonic detection and applying conditions. They also have some weaknesses, such as high coupling requirements between sensors and the surface of the container wall. In actual measurement, an appropriate coupling agent should be chosen according to the material of the container, and the thickness of the coupling layer should be adjusted according to experimental results so that the incident ultrasound waves are consistent throughout the measuring process, because the premise is to determine the liquid level by comparing the impedance characteristics of different positions. Otherwise, the measuring result is inaccurate or meaningless.

When a beam of ultrasound is transmitted from above and below the liquid level respectively, the two reflected echo energies are significantly different because of the different ultrasonic impedance between gas and liquid media in a container. This study presents a novel measurement method. The proposed method uses the balance of echo energy received by two sensors to determine the liquid level from the outside of a sealed container. It can solve the problems caused by bad coupling between sensors and the container wall, and can enhance the stability and reliability of measurement.

**Figure 1:**
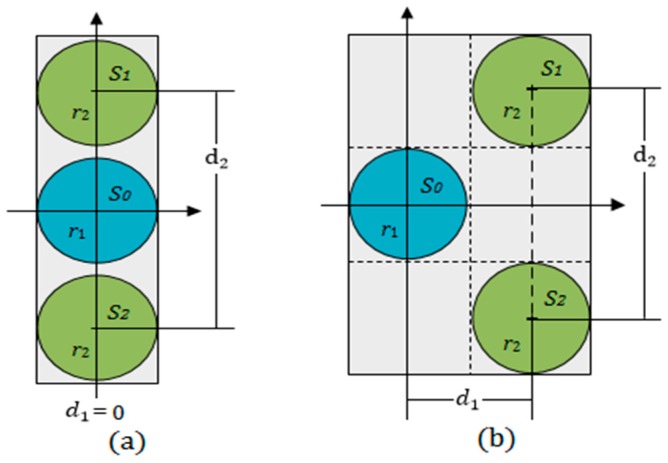
The arrangement rules of three sensors: (**a**) $d_{1}=0$; and (**b**) $d_{1}=r_{1}+r_{2}$.

In the proposed method, three round plate ultrasonic sensors are used and arranged as shown Figure 1, and meet the following conditions:

- The transducer $S_{0}$ is used as an ultrasonic transmitter. The other two sensors, $S_{1}$ and $S_{2}$, are used as receivers;
- The distances $d_{1}$ and $d_{2}$ meet the conditions $0\leq d_{1}\leq r_{1}+r_{2}$, $2r_{2}\leq d_{2}\leq \left(4r_{1}+2r_{2}\right)$. The two receiving sensors $S_{1}$ and $S_{2}$ are arranged symmetrically along the horizontal coordinate axis;
- Three sensors are placed on the same coupling plane and encapsulated in a rectangular plastic box with epoxy;
- In the detection process, the sensors are moved along the longitudinal direction on the surface of a container wall.

**Figure 2:**
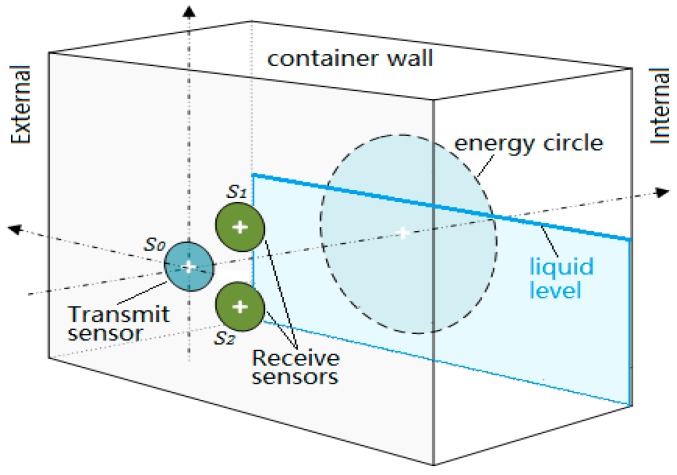
The measuring principle of the proposed method.

Measuring principle: As shown in Figure 2, when using the transducer $S_{0}$ to emit a beam of ultrasonic waves perpendicularly to the outer surface of a container wall, both of the receiving sensors $S_{1}$ and $S_{2}$ could detect echoes reflected by the inner surface if the wall thickness L is less than the length of the sound field. Because they are geometrically symmetrical to the transmitting transducer $S_{0}$, if the reflection boundary conditions at the inner surface are consistent, the echo energy received by the two receiving sensors should be equal in magnitude. The different ultrasonic impedance between the gas and liquid media will make the reflection and transmission of sound waves different at the inner surface. Therefore, the acoustic boundary conditions will be changed continuously when the transmitting transducer $S_{0}$ is moved near the liquid level, and the balance of echo energy received by the two receiving sensors $S_{1}$ and $S_{2}$ will be destroyed. In this research, the balance is used to determine the liquid level.

Advantages: In the proposed method, the two receiving sensors are arranged along the same coupling plane, and the balance of echo energy received by the two receiving sensors is the only thing that needs attention. In other words, the ratio of two received echo energy at the same position is what we care about, rather than the magnitude of echo energy value. The proposed method reduces the coupling requirements of transmitting sensor, and enhances the reliability, stability, and sensitivity of detection.

## 2. Theory and Methods

### 2.1. The Energy Circle

**Figure 3:**
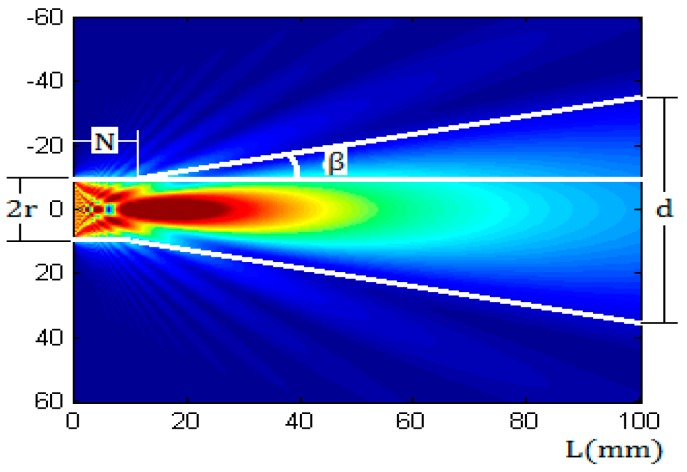
The sound field of a 1 MHz, 20 mm diameter round piston transducer in an aluminum alloy as calculated with a Multi-Gaussian Beam Model.

According to the model and measurement of Schmerr [7,8], the sound field of a round piston sensor in a solid medium has two different parts [9,10]. In the near field, the beam can maintain a cylindrical shape to transmit because of its smaller divergence angle. In the far field region, the beam propagates with a certain divergence angle, as shown in Figure 3.

The sound field was calculated by using Multi-Gaussian Beam Model [11,12,13], which can simulate the sound field of a transducer in 2D and 3D. The ultrasonic wave frequency was 1 MHz, the diameter of the transducer was 20 mm, the wall material was aluminum, in which the compressional wave speed was 6300 m/s, the shear wave speed was 3100 m/s, and the ultrasonic impedance was 17 × 10^5^ gm/cm^2^·s. The length of the near field N and the diffusion angle β are given by Equations (1) and (2), respectively [14].

(1) $$N=\frac{r^{2}}{\lambda_{c}}$$

(2) $$\beta =\arcsin\frac{1.22\lambda_{c}}{2r}$$

where $\lambda_{c}$ is the wavelength of ultrasonic waves in a metal wall, r is the radius of the sensor.

From Figure 3, it can be inferred that the ultrasonic beam will form a circular region on inner surface of a container wall after a propagating distance, and the beam energy is mainly concentrated in this region. The projected circular region is referred to as the energy circle, of which the diameter is expressed by d and can be calculated by Equation (3):

(3) $$\{\begin{array}{c} d=2r\;\left(L\leq N\right)\\ d=2\left[r+\left(L-N\right)\tan\beta \right]\;(L>N) \end{array}$$

### 2.2. Sound Pressure Distribution at Any Point outside the Axis

**Figure 4:**
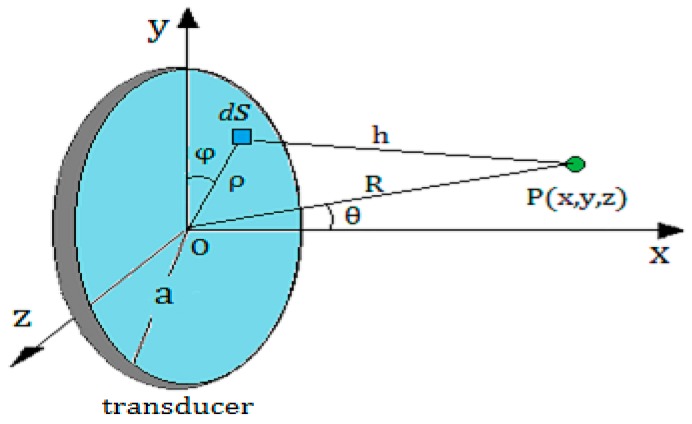
Calculating the acoustic field characteristics of a round piston transducer at any point outside the axis according to the Kirchhoff integral theorem.

As shown in Figure 4, P(x,y,z) is a point outside the axis of the round piston sensor, the distance from the center O(0,0,0) of the sensor to the point P(x,y,z) is marked by R=D(O,P). The angle between R and the x-axis is denoted by θ. Then, according to the Kirchhoff integral theorem [15,16,17], the sound pressure at the point P can be calculated as

(4) $$p\left(R,\theta \right)=\left(\frac{{\pi a}^{2}}{\lambda R}\right)\left[\frac{2J_{1}\left(\mathrm{kasin}\theta \right)}{\mathrm{kasin}\theta }\right]\cdot p_{0}$$

where $p_{0}$ is the initial sound pressure of the sound source, λ is the wavelength of ultrasonic waves in a medium, a is the radius of the sensor, and k is the wave number; $J_{1}$ is the first kind of first order Bessel function. The geometric meaning of other variables is shown in Figure 4.

Because of the interference in the near field of a round piston transducer, Equation (4) is valid only in the far field, which requires the wall thickness to meet the condition L>N in a detection. This requirement can be achieved by adjusting the near field length N for a given container. Equation (1) demonstrates that the radius of the transducer and the ultrasound wave length in a container wall are critical factors, and the latter is associated with the transmitting frequency and the wall material.

### 2.3. Analysis of Echo Energy

According to the basic knowledge of acoustics, ultrasonic waves will be refracted and reflected at an interface with discontinuous impedance, which follows the refraction and reflection principle of sound waves.

**Figure 5:**
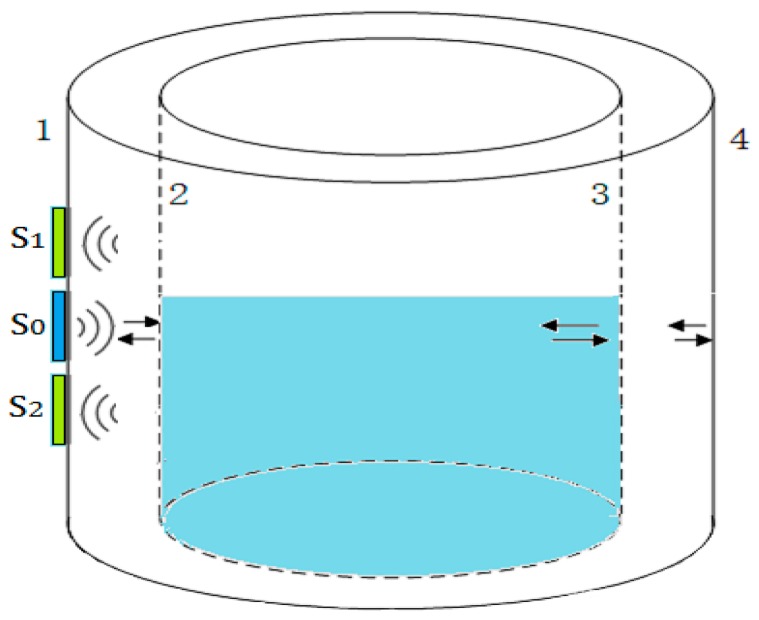
The process of an ultrasonic beam propagating in a container. $S_{0}$ is a transmitter, $S_{1}$ and $S_{2}$ are two receivers, 1,2,3,4 represent the four interfaces of the container.

As shown in Figure 5, when the transmitting transducer $S_{0}$ is excited to transmit an ultrasonic beam into the wall, part of the ultrasonic beam will be reflected by the interface 2, and the echoes will be oscillated repeatedly between interfaces 1 and 2 until they decay to zero. Another part of the beam will transmit into the gas-liquid medium in container, of which the energy may decay to zero in propagating process, or may penetrate the gas-liquid medium and be reflected by interfaces 3 and 4. All of these reflected echoes can be detected by the two receiving sensors $S_{1}$ and $S_{2}$ that are attached to the outer surface of the container wall.

**Figure 6:**
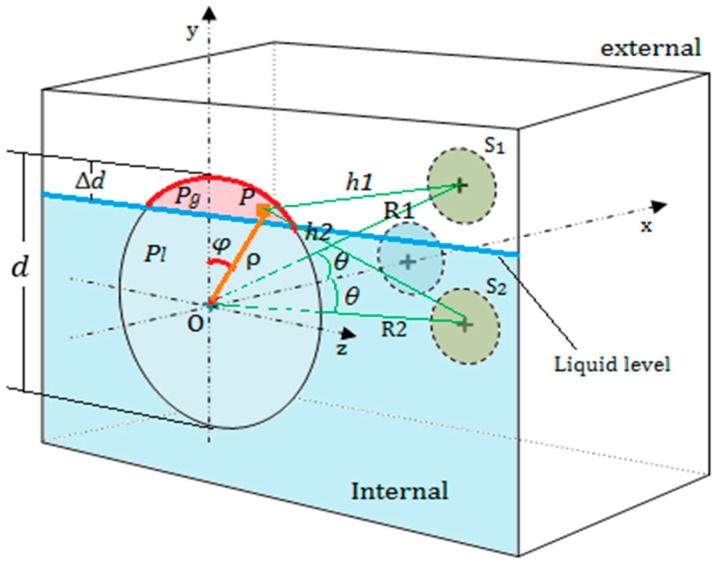
Calculating the echo energy received by the two receiving sensors $S_{1}$ and $S_{2}$.

As shown in Figure 6, in the detection process, when the top of the energy circle exceeds the liquid level, the exceeding height is represented by Δd and 0≤Δd≤d. Assuming that the total area of the energy circle is A, the area of the energy circle above the liquid level is denoted by $A_{T}$, and let $r_{s}=A_{T}/A$.

In Figure 6, when taking ρ=d/2 and 0≤φ≤π, we can get the value of Δd and the ratio $r_{s}$ by Equations (5) and (6).

(5) $$\Delta d=\frac{d}{2}\left(1-\cos\varphi \right)$$

(6) $$r_{s}=\frac{1}{\pi }\left(\varphi -\sin\varphi \cos\varphi \right)$$

When 0≤Δd≤d, the energy circle is divided into two parts by the liquid level, which makes the acoustic boundary conditions of the two parts different. When an ultrasonic beam propagates first to interface 2, the sound pressure in two parts of energy circle are represented by $P_{g}$ and $P_{l}$ receptively, and assuming $P_{g}>P_{l}$, as shown in Figure 6.

Here, the energy circle can be approximately regarded as a round transmitting transducer. Assuming that there is a point P(ρ,φ) in the upper part of the energy circle, and with the energy circle moving up, the average sound pressure at the point P increases from $P_{l}$ to $P_{g}$. According to Equation (4), the sound pressure of the two receiving sensors $S_{1}$ and $S_{2}$ can be obtained by integrating all the points in the red part of the energy circle, which can be described approximately by Equations (7) and (8)

(7) $$\Delta p_{s1}\left(h1,\theta \right)=\left(\frac{\pi {\left(d/2\right)}^{2}\cdot r_{s}}{\lambda h1}\right)\left[\frac{2J_{1}\left(k\left(d/2\right)\sin\theta \right)}{k\left(d/2\right)\sin\theta }\right]\cdot \left(p_{g}-p_{l}\right)\cdot {\pi r}_{2}^{2}$$

(8) $$\Delta p_{s2}\left(h2,\theta \right)=\left(\frac{\pi {\left(d/2\right)}^{2}\cdot r_{s}}{\lambda h2}\right)\left[\frac{2J_{1}\left(k\left(d/2\right)\sin\theta \right)}{k\left(d/2\right)\sin\theta }\right]\cdot \left(p_{g}-p_{l}\right)\cdot {\pi r}_{2}^{2}$$

where ρ is the polar radius, φ is the polar angle, θ is the angle between R1 (or R2) and the x-axis, $h_{1}=D\left(P,S_{1}\right)$, $h_{2}=D\left(P,S_{2}\right)$, $R1=D\left(O,S_{1}\right)=h_{1}^{2}+\rho^{2}-2\sin\theta \cos\varphi$, $R2=D\left(O,S_{2}\right)=h_{2}^{2}+\rho^{2}-2\sin\theta \cos\varphi$, $P_{g}=P_{0}e^{-\alpha L}R_{\mathrm{mg}}$ and $P_{l}=P_{0}e^{-\alpha L}R_{\mathrm{ml}}$, $R_{\mathrm{mg}}$ represents the reflection coefficient at the upper part of the energy circle, $R_{\mathrm{ml}}$ represents the reflection coefficient at the lower part of the energy circle.

Furthermore, it is assumed that the echo energy in the wall will decay to a very small amount after n times, which can be negligible relative to the total energy received by receiving sensor. Therefore, when the sound beam is reflected to the outer surface of the wall at the n times, the total pressure of $S_{1}$ and $S_{2}$ can be derived as following equations:

(9) $$\sum p_{s1}\left(h1,\theta \right)=\left(\frac{\pi {\left(\frac{d}{2}\right)}^{2}}{\lambda h1}\right)\left[\frac{2J_{1}\left(k\left(d/2\right)\sin\theta \right)}{k\left(d/2\right)\sin\theta }\right]\cdot P_{0}\cdot {\pi r}_{2}^{2}\cdot \left(r_{s}\cdot \overset{n}{\underset{i=1}{\sum }}R_{\mathrm{mg}}^{i}R_{\mathrm{ma}}^{i-1}e^{-2i\alpha L}+(1-r_{s})\cdot \overset{n}{\underset{i=1}{\sum }}R_{\mathrm{ml}}^{i}R_{\mathrm{ma}}^{i-1}e^{-2i\alpha L}\right)$$

(10) $$\sum p_{s2}\left(h2,\theta \right)=\left(\frac{\pi {\left(d/2\right)}^{2}}{\lambda h2}\right)\left[\frac{2J_{1}\left(k\left(d/2\right)\sin\theta \right)}{k\left(d/2\right)\sin\theta }\right]\cdot P_{0}\cdot {\pi r}_{2}^{2}\cdot \left(r_{s}\cdot \overset{n}{\underset{i=1}{\sum }}R_{\mathrm{mg}}^{i}R_{\mathrm{ma}}^{i-1}e^{-2i\alpha L}+(1-r_{s})\cdot \overset{n}{\underset{i=1}{\sum }}R_{\mathrm{ml}}^{i}R_{\mathrm{ma}}^{i-1}e^{-2i\alpha L}\right)$$

where $R_{\mathrm{ma}}$ represents the reflection coefficient at interface 1, α is the attenuation coefficient of a container, and L is the thickness of a container wall.

In conclusion, when the transmitting transducer $S_{0}$ and the two receiving sensors $S_{1}$ and $S_{2}$ are arranged by the rules in Figure 1, near the liquid level, the echo energy received by two receiving sensors will be changed respectively, because the reflection boundary conditions of the energy circle are changed. The balance of the echo energy between the two receiving sensors can be used to determine the liquid level.

## 3. Experimental Results 

### 3.1. Measurement System and Initial Conditions

**Figure 7:**
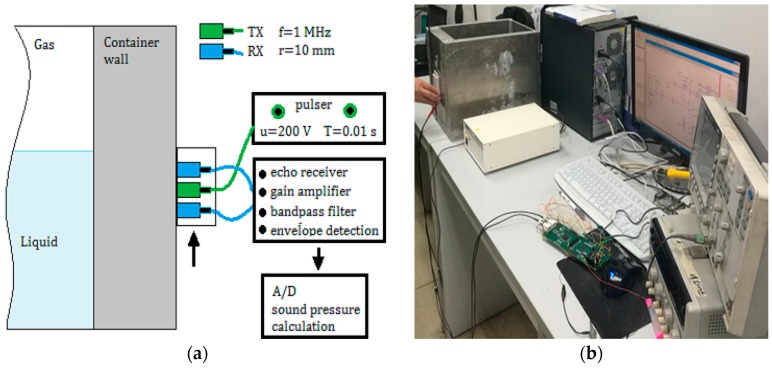
Measurement system: (**a**) TX is a transmitting transducer, RX is a receiving sensor; and (**b**) calibration device in the experiment.

The experiment system and the calibration device are shown in Figure 7. In the evaluation of the proposed method, an aluminum container with different wall thickness was used, in which the liquid media was water and the gaseous media was air. The initial conditions and initial values of the parameters used in this study are shown in Table 1. In order to simplify the discussion process, the radiuses of the transmitting transducer and two receiving sensors were chosen as the same value. 

### 3.2. Results of Experiment

#### 3.2.1. Comparison of Echo Energy under Different Coupling

**Figure 8:**
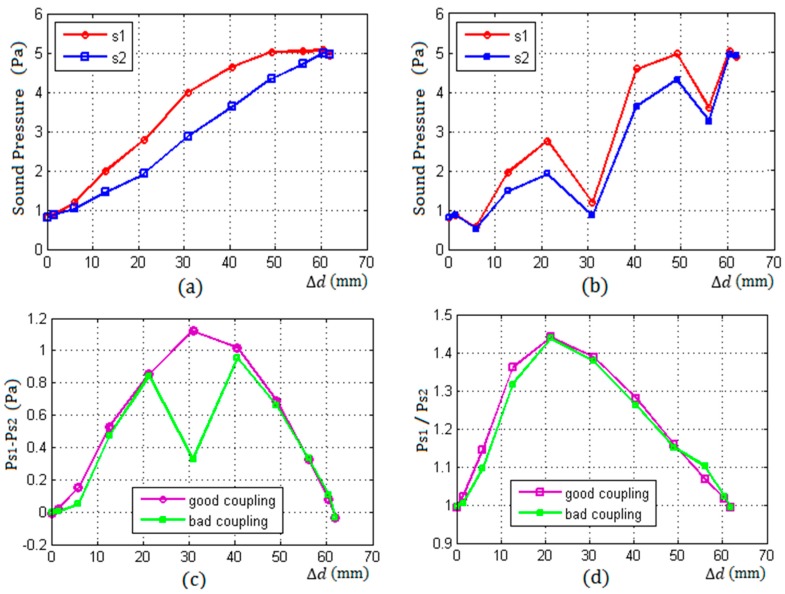
The result of two different coupling as the thickness of wall L = 50 mm, (**a**) the curves of sound pressure of $S_{1}$ and $S_{2}$ with a good coupling; (**b**) the curves of sound pressure of $S_{1}$ and $S_{2}$ with a bad coupling; (**c**) the difference of sound pressure of $S_{1}$ and $S_{2}$ under two different couplings; and (**d**) the ratio of sound pressure of $S_{1}$ and $S_{2}$ under two different couplings.

Figure 8 shows the measurement results with the thickness of a container wall being 50 mm.

Figure 8a,b shows the change of echo pressure received by the two receiving sensors $S_{1}$ and $S_{2}$ with the increase of Δd from 0 to d under a good coupling and a bad coupling conditions respectively.

From Figure 8a, under good coupling conditions, it can be seen that both of the received energy of the two sensors $S_{1}$ and $S_{2}$ increased with the increase of Δd from 0 to d, and the two increments of the sensors $S_{1}$ and $S_{2}$ were not equal at the same position with the same Δd. When Δd≥d and Δd≤0, the state of the two receiving sensors was balance, because the received echo energy of $S_{1}$ and $S_{2}$ were equal. 

Figure 8b shows that both of the echo energy of $S_{1}$ and $S_{2}$ significantly fluctuated under bad coupling condition at some positions, but it also can be seen that the changing directions of the two energy were consistent: both of them increased or decreased at the same position, because the two receiving sensors had the same coupling characteristic.

Figure 8c,d shows the difference and ratio of the two echo energy of $S_{1}$ and $S_{2}$ with the increase of Δd from 0 to d under two different conditions respectively. From Figure 8d, it can be seen that the ratio of the two echo energy received by the receiving sensors $S_{1}$ and $S_{2}$ were essentially changeless and did not appear to fluctuate, whether the coupling between the sensors and the wall was good or bad.

#### 3.2.2. Results under Two Different Arrangements of Sensors

In Table 2, the data presented are the average values of the results of three times measurements. The symbol $h_{l}$ represents the actual height of the liquid level in a container, $\bar{h_{m}}$ is the average measuring result of the proposed method, and $\bar{\Delta E}$ is the average error. 

Table 2 shows the measurement results under two different arrangements as described in Figure 1. In the first type of rules, $d_{1}=0$, $d_{2}$ were taken as 4r, 5r, 6r, 8r, and 10r respectively. In the second type of rules, $d_{1}=2r$, $d_{2}$ were taken as 2r, 3r, 4r, 6r, and 8r respectively.

**Figure 9:**
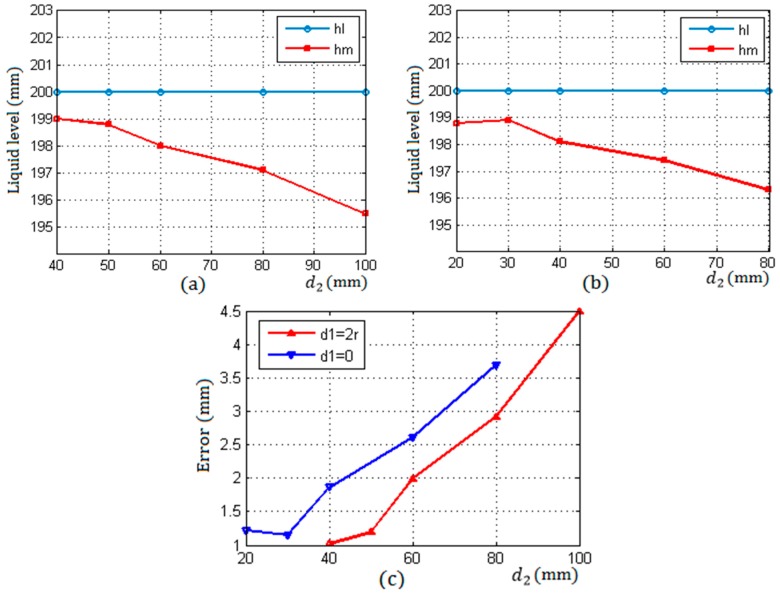
The measuring results under two different arrangements. (**a**) $d_{1}=0$ and $d_{2}=\left(4\sim 10\right)r$; (**b**) $d_{1}=2r$ and $d_{2}=\left(2\sim 8\right)r$; (**c**) errors.

Figure 9a corresponds to the first type of rules and shows that the measurement accuracy decreased gradually with the increase of $d_{2}$ from 4r to 10r. This is because the echo energy received by the two receiving sensors gradually reduced with the increase of $d_{2}$ which resulted in a reduction of resolution. Therefore, in this arrangement, when the distance $d_{2}$ between $S_{1}$ and $S_{2}$ is taken as the minimum value 4r, the measurement accuracy is optimal.

**Figure 10:**
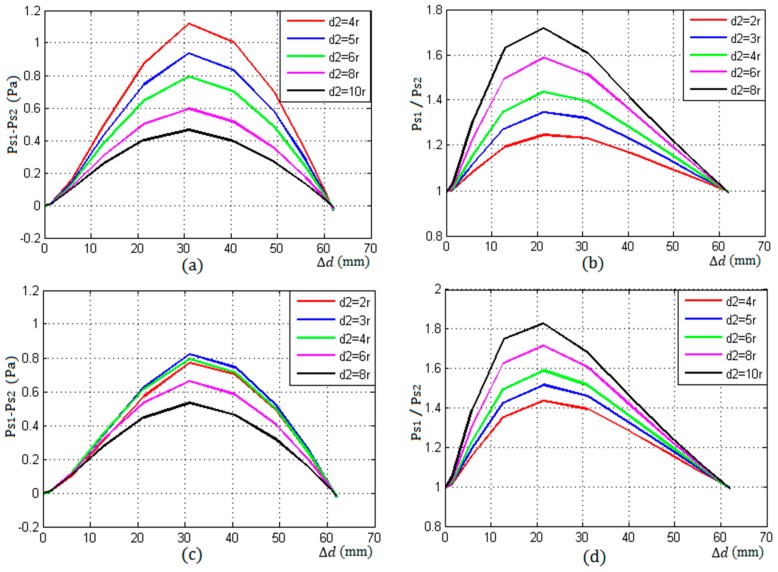
The difference and ratio of sound pressure of $S_{1}$ and $S_{2}$ under two different arrangements. (**a**,**b**) $d_{1}=0$, $d_{2}=\left(4\sim 10\right)r$; (**c**,**d**) $d_{1}=2r$, $d_{2}=\left(2\sim 8\right)r$.

Figure 9b corresponds to the second type of rules in this arrangement, when $d_{2}$ is taken as the minimum value 2r, the measurement accuracy is not optimal, which is different from the first rule. It can be seen that when the distance between $S_{1}$ and $S_{2}$ was taken as a minimum $d_{2}=2r$, the difference of the two energy of $S_{1}$ and $S_{2}$ was less than that of $d_{2}=3r$ and $d_{2}=4r$ as shown in Figure 10c.

Figure 9c shows the errors with the increase of the distance $d_{2}$ between $S_{1}$ and $S_{2}$ under two different arrangement rules. When $d_{1}=0$, $d_{2}=4r$ in the first rule and $d_{1}=2r$, $d_{2}=3r$ in the second arrangement, the measurement accuracy was optimal, reaching about 1mm, which was higher than that of the method in literatures [1,2]. In addition, compared with the methods mentioned in the introduction, the proposed method has higher stability and reliability.

The uncertainty of the proposed method was the difference of ultrasonic impedance between gas and liquid in containers. If this difference is so small that two parts of echo energy are quite similar to each other, as the sensors are, respectively, above and below the liquid level, and the measurement will not be possible.

Figure 10a,b show the difference and ratio of the echo energy of $S_{1}$ and $S_{2}$ change with the increase $d_{2}$ from 4r to 10r, in the first type of arrangement with a good coupling. From them, it can be seen that the curve values of the difference become smaller, the ratio becomes bigger with the increase of $d_{2}$, and the detection resolution reduced gradually.

Figure 10c,d shows the difference and ratio of the echo energy of $S_{1}$ and $S_{2}$ change with the increase $d_{2}$ from 2r to 8r, in the second type of arrangement with a good coupling. From Figure 10c, it can be seen that the values of the difference was the maximum when $d_{2}=3r$ rather than $d_{2}=2r$. when $d_{2}>4r$, the difference decreased with the increase of $d_{2}$. From Figure 10d, it can be seen that the curve values of the ratio became bigger with the increase of $d_{2}$.

## 4. Discussion

According to this study, the detection results are affected by the rules of sensors arrangement, which is determined by the values of $d_{1}$ and $d_{2}$. Another important influence factor is the thickness L of the container wall, which is similar to $d_{2}$. From experimental result, it can be known that with the increase of the thickness L, the echo energy received by two receiving sensors will decrease and cause a reduction in measurement resolution. On the other hand, if increasing the incident frequency or the size of the transmitting sensor, the accuracy problem caused by the increase of the thickness of the wall can be improved.

In this study, two kinds of special arrangement rules are used in the experiment, and their measurement results are discussed under different coupling conditions. In actual detection, according to the different detection environment and initial conditions, the optimal sensors can be combined by the arrangement rules and requirements as shown in Figure 1.

## 5. Conclusions

The experimental results show that the proposed method is an effective and nondestructive ultrasonic method for liquid level measurement, which has higher detection accuracy, reliability and stability, and has higher practical value.

The proposed method reduces the coupling requirements between the sensors and the container wall, which makes it unnecessary for the energy of incident beam to be maintained in a very stable state during the whole detection process. Therefore, the operation of the proposed method is more convenient and flexible, the detection process is more easily controlled, and the measurement results are more reliable, stable, and accurate than the previously developed methods mentioned in the introduction. 

## Figures and Tables

**Table 1:** Initial values of the experimental parameters.

Parameters Meaning	Initial Values
the thickness of the container wall	L = 8 mm, 25 mm, 40 mm, 50 mm
the impedance of the metal container	$Z_{m}=17\times 10^{5}\;\mathrm{gm}/{\mathrm{cm}}^{2}\cdot s$
the impedance of gas media in the container	$Z_{g}=0.0004\times 10^{5}\;\mathrm{gm}/{\mathrm{cm}}^{2}\cdot s$
the impedance of liquid media in the container	$Z_{l}=1.48\times 10^{5}\;\mathrm{gm}/{\mathrm{cm}}^{2}\cdot s$
the reflection coefficient between the inner wall and gas	$R_{\mathrm{mg}}=0.99995294$
the reflection coefficient between the inner wall and liquid	$R_{\mathrm{ml}}=0.83982683$
the reflection coefficient between the outer wall and air	$R_{\mathrm{ma}}=0.99995294$
the center frequency of the transmitting transducer	$f_{c}=1\;\mathrm{MHz}$
the repetition frequency of a pulse	$f_{r}=100\;\mathrm{Hz}$
the repetition period of a pulse	T=0.01 s
the excitation voltage	U=200 V
the operating temperature range of sensors	(−10~80) °C
the diameter of the sensors	$r=r_{1}=r_{2}=10\;\mathrm{mm}$
the ultrasonic attenuation coefficient in the container wall.	α=2 dB/m

**Table 2:** The result of measurement corresponding to the two arrangement in Figure 1 (mm).

L	r1,r2	N	d	d1	d2	$\bar{h_{m}}$	$h_{l}$	$\bar{\Delta E}$
50	10	12.5	61.93	0	4r	198.99	200	1.02
50	10	12.5	61.93	0	5r	198.82	200	1.19
50	10	12.5	61.93	0	6r	198.01	200	1.99
50	10	12.5	61.93	0	8r	197.08	200	2.92
50	10	12.5	61.93	0	10r	195.52	200	4.49
50	10	12.5	61.93	2r	2r	198.78	200	1.22
50	10	12.5	61.93	2r	3r	198.85	200	1.15
50	10	12.5	61.93	2r	4r	198.14	200	1.86
50	10	12.5	61.93	2r	6r	197.39	200	2.61
50	10	12.5	61.93	2r	8r	196.31	200	3.69
